# Introduction of Lazy Luna an automatic software-driven multilevel comparison of ventricular function quantification in cardiovascular magnetic resonance imaging

**DOI:** 10.1038/s41598-022-10464-w

**Published:** 2022-04-22

**Authors:** Thomas Hadler, Jens Wetzl, Steffen Lange, Christian Geppert, Max Fenski, Endri Abazi, Jan Gröschel, Clemens Ammann, Felix Wenson, Agnieszka Töpper, Sascha Däuber, Jeanette Schulz-Menger

**Affiliations:** 1 Charité – Universitätsmedizin Berlin, corporate member of Freie Universität Berlin and Humboldt-Universität Zu Berlin, Berlin, Germany; 2 Working Group On CMR, Experimental and Clinical Research Center, a cooperation between the Max-Delbrück-Center for Molecular Medicine in the Helmholtz Association and the Charité - Universitätsmedizin Berlin, Berlin, Germany; 3 DZHK (German Centre for Cardiovascular Research), partner site Berlin, Berlin, Germany; 4 Department of Cardiology and Nephrology, HELIOS Hospital Berlin-Buch, Berlin, Germany; 5 Siemens Healthineers, Erlangen, Germany; 6 Department of Computer Sciences, Hochschule Darmstadt - University of Applied Sciences, Darmstadt, Germany; 7 Department of Internal Medicine III, Cardiology, Lutherstadt Wittenberg, Evangelisches Krankenhaus Paul Gerhardt Stift, Wittenberg, Germany

**Keywords:** Quality control, Software, Cardiology

## Abstract

Cardiovascular magnetic resonance imaging is the gold standard for cardiac function assessment. Quantification of clinical results (CR) requires precise segmentation. Clinicians statistically compare CRs to ensure reproducibility. Convolutional Neural Network developers compare their results via metrics. Aim: Introducing software capable of automatic multilevel comparison. A multilevel analysis covering segmentations and CRs builds on a generic software backend. Metrics and CRs are calculated with geometric accuracy. Segmentations and CRs are connected to track errors and their effects. An interactive GUI makes the software accessible to different users. The software’s multilevel comparison was tested on a use case based on cardiac function assessment. The software shows good reader agreement in CRs and segmentation metrics (Dice > 90%). Decomposing differences by cardiac position revealed excellent agreement in midventricular slices: > 90% but poorer segmentations in apical (> 71%) and basal slices (> 74%). Further decomposition by contour type locates the largest millilitre differences in the basal right cavity (> 3 ml). Visual inspection shows these differences being caused by different basal slice choices. The software illuminated reader differences on several levels. Producing spreadsheets and figures concerning metric values and CR differences was automated. A multilevel reader comparison is feasible and extendable to other cardiac structures in the future.

## Introduction

Non-invasive imaging techniques such as Cardiovascular Magnetic Resonance (CMR) have become prominent in research and medical practice in the cardiovascular field^1^. CMR is accepted as the gold standard in several applications, such as biventricular function assessment. Echocardiography remains the first-line method in clinical routine for function assessment, but CMR is increasingly listed in guidelines of the European Society of Cardiology^2^ as the back-up method. CMR offers quantification of cardiac function, volume and mass for the left and right ventricle (LV, RV). Volumes include the end-systolic, end-diastolic and the stroke volume (ESV, EDV, SV). Function means the ejection fraction (EF) whereas the mass refers to the myocardial mass. Calculating these values requires a reproducible and precise segmentation of the LV and RV cavities as well as the LV myocardium.

In clinical practice as well as in research, readers annotate contours often in accordance with the SCMR guidelines^1^. However, manual segmentation is time-consuming and remains prone to inter- and intraobserver variability^3,4^. In order to characterize pathologies with diagnostic approaches, inter- and intraobserver analyses are performed in order to ensure the methods’ statistical reproducibility and accuracy^3–6^. Segmentations are based on subpixel resolution producing contours as polygons^1^. An objective analysis of segmentation differences could be based on segmentation metrics such as the Dice Similarity Coefficient (Dice) or the Hausdorff Distance (HD) as typically used in computer vision challenges and tasks^7–9^. Metrics are typically not used to compare segmentation similarity in context to clinical relevance and decision-making.

In recent years several convolutional neural network (CNN) developers have trained CNNs to contour CMR-images similar to medical experts^9–14^. The annotations are generated in a fraction of the time a reader would require and are often performed on subpixel resolution as segmentation masks^9,13–15^. CNNs demonstrate promising clinical results within the variability of interobserver errors^16,17^, while still making human atypical mistakes^18–20^. Segmentation metrics (such as the Dice and HD) are typically used to compare CNNs to medical readers on the level of individual segmentations^9,16,21^. The qualitative nature of the human atypical segmentation differences remains elusive^18,20^.

The goal of this paper is to design software that is capable of an automatic multilevel reader comparison. Usability by CNN developers as backend software and by medical experts as a graphical user interface (GUI) should be given alike.

## Methods

The software Lazy Luna was designed to offer a multilevel reader comparison that covers segmentations and CRs. Metrics and CRs are calculated accurately. Segmentations and CRs are connected to allow for error tracking. An interactive GUI makes the software accessible to clinical readers and CNN developers. Lazy Luna’s functionality was demonstrated by performing a multilevel interobserver analysis.

### Data

The dataset encompasses short-axis balanced steady-state free precession (bSSFP) cine CMR images of 13 patients (39 ± 13 years, 7/6 male/female). They were produced on a 1.5 T Avanto fit, Siemens Healthineers. The cases were selected randomly from an on-going trial. The central criterion was the performance of an interobserver analysis of the right and left ventricle. A short image stack consists of 16–18 slices and 30 phases. Two expert readers segmented the images using Circle Cardiovascular Imaging: cvi42 version 5.12.1.^22^. They segmented the LV and RV cavity and contoured the LV myocardium and papillary muscles.

The local ethics committee of Charité Medical University Berlin gave ethics approval for the original study (approval number EA1/198/20). All patients gave their written informed consent before participating in the study. All methods were carried out in accordance with relevant guidelines and regulations.

### Cases

**Figure 1:**
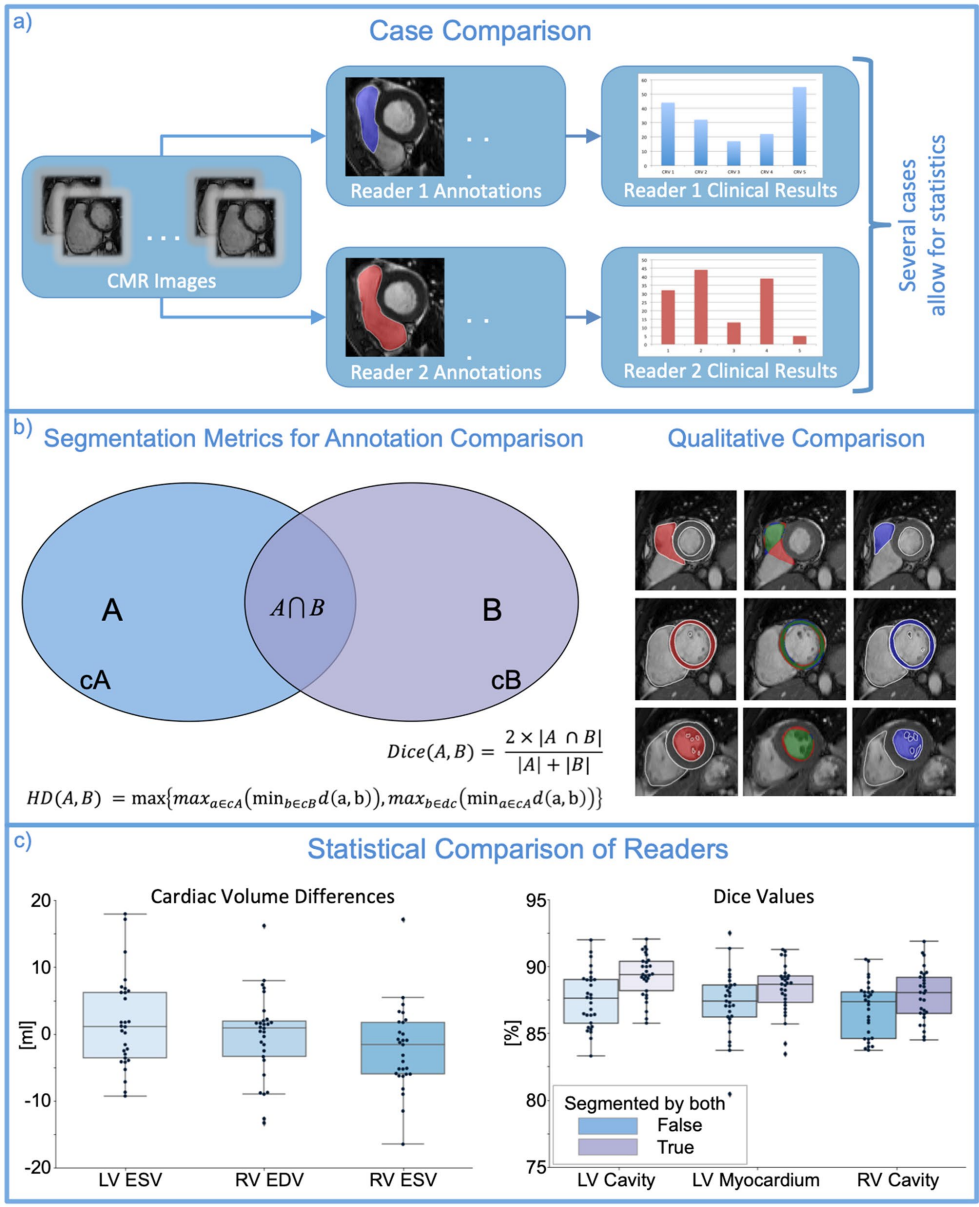
Multilevel Reader Comparison. Caption: At the top (**a**) a case comparison is presented. Comparable cases concern CMR images that were segmented by two different readers. Clinical results are generated from images and segmentations. Segmentation metrics (**b**), such as Dice and Hausdorff metric, provide quantitative comparisons of segmentations. Next to the metrics, qualitative visualizations of segmentation differences are presented. The first reader’s segmentation is coloured blue, the second red and their agreement in green. Reader comparisons are modelled as the distributions of clinical result differences and metric value distributions. LV: Left ventricle, RV: Right ventricle, ESV: End-systolic volume, EDV: End-diastolic volume, cA (cB): Contour of Area A, ml: Millilitre, HD: Hausdorff metric.

Cases contain images, annotations (i.e. segmentations, points, etc.) of these images and clinical values that were calculated on the basis of these images and their annotations (Fig. 1a). The images were sorted into phases and slices. Two cases can be compared to each other when they reference the same original images. When many comparable cases were segmented by two readers statistics can be performed on the metric values and CRs (Fig. 1).

The images, segmentations and CRs refer to the same case allowing for tracking the effect of failed segmentations on differences in assessed CRs. For many comparable cases the outliers of CRs can be identified and the causes for their particularity backtracked to their origin in specific contours and their cardiac position (i.e. basal, midventricular, apical).

The images are stored in the Dicom^23^ (Digital Imaging and Communications in Medicine) format. Dicom images are used to store images as well as information pertaining to those images. The images are loaded using the Python package Pydicom^24^. Annotations are stored in a custom Lazy Luna format, as pickle files containing a Python dictionary that maps contour names to Shapely^25^ objects. Shapely is described in “Geometrical representation and metrics”.

### Data pre-processing

Lazy Luna was designed to emphasize precision. The analysis tool can only be applied if the user transforms images and annotations to fit Lazy Luna’s interface. Lazy Luna requires images in Dicom format and annotations as pickle-files containing Shapely objects. Thus, pre-processing the data is a requirement for using the tool. An easy to use Data Pre-processing GUI for labelling Dicom images as well as linking the images to segmentations was used.

Finding the short-axis cine Dicom images in a set of several thousand images is an error-prone task and user intervention is essential. Images are manually identified as short-axis cine images by adding a Lazy Luna Dicom tag. The clinicians contoured the relevant images and stored the contours as workspaces. These workspaces were converted into the custom Lazy Luna annotation format.

### Geometrical representation and metrics

Lazy Luna uses Shapely to process annotations. Shapely is a Python package for manipulating and analysing geometric objects (i.e. polygons, lines, points)^25^. Segmentations are modelled as polygons (LV, RV endocardial contour and LV myocardium) or MultiPolygons (papillary muscles). Shapely is capable of performing a wide array of precise geometrical operations, such as area calculation, intersection, union and calculating the Hausdorff distance (HD)^26^. The Dice metric is calculated using intersection and union operations on two Shapely objects (Fig. 1b). The millilitres and their differences (ml Diff) are calculated using Dicom tag information on pixel height, width and slice thickness in mm:

$$ ml\, Diff\left( {A,B} \right) = \left( {\left| A \right| - \left| B \right|} \right) \times\, area \,per \,pixel\, \times \,slice\, thickness $$

$$ Dice\left( {A,B} \right) = \frac{{2 \times \left| {A \cap B} \right|}}{\left| A \right| + \left| B \right|} $$

$$ HD\left( {A,B} \right) = {\text{max}}\left\{ { max_{a \in cA} \left( {{\text{min}}_{b \in cB} d\left( {{\text{a}},{\text{b}}} \right)} \right), max_{b \in dc} \left( {{\text{min}}_{a \in cA} d\left( {{\text{a}},{\text{b}}} \right)} \right) } \right\} $$

We offer two different averages for the Dice metric. The first one averages over all images, the second only over images segmented by both readers. The first rewards correct segmentation decisions, e.g. if the CNN should not and does not segment an image it considers this as an example of 100% Dice. If it makes an incorrect segmentation decision then it considers this mistake as 0% Dice. The second Dice average only considers the segmentation similarity for segmented images and discounts the relevance of the segmentation decision. It exclusively reflects the similarity of segmentation areas.

In order to calculate precise values for segmentation masks (typical outputs of CNNs) these must also be converted to Shapely objects. The transformation method should outline the pixelated segmentation mask precisely. For example, Rasterio’s rasterize function can be used to produce outlines of segmentation masks in Shapely format^27^.

### Software conception

The software Lazy Luna builds on several implemented classes following the object oriented programming paradigm. Classes are indicated with a capital letter. The Cases described above are a container class for images and annotations. An Annotation Type (i.e. segmentations of short-axis cine images) can be attached to a case and offers several visualization functions as well as geometric operations. Categories can be attached to a case in order to structure the case’s images into slices and phases by using Dicom image information. Categories identify relevant phases for Clinical Results. Clinical Result classes can be attached to a Case in order to calculate CRs based on the images, annotations and categories. Case Comparisons contain two cases that reference the same images. Metrics can be attached to a Case Comparison to calculate metric values.

**Figure 2:**
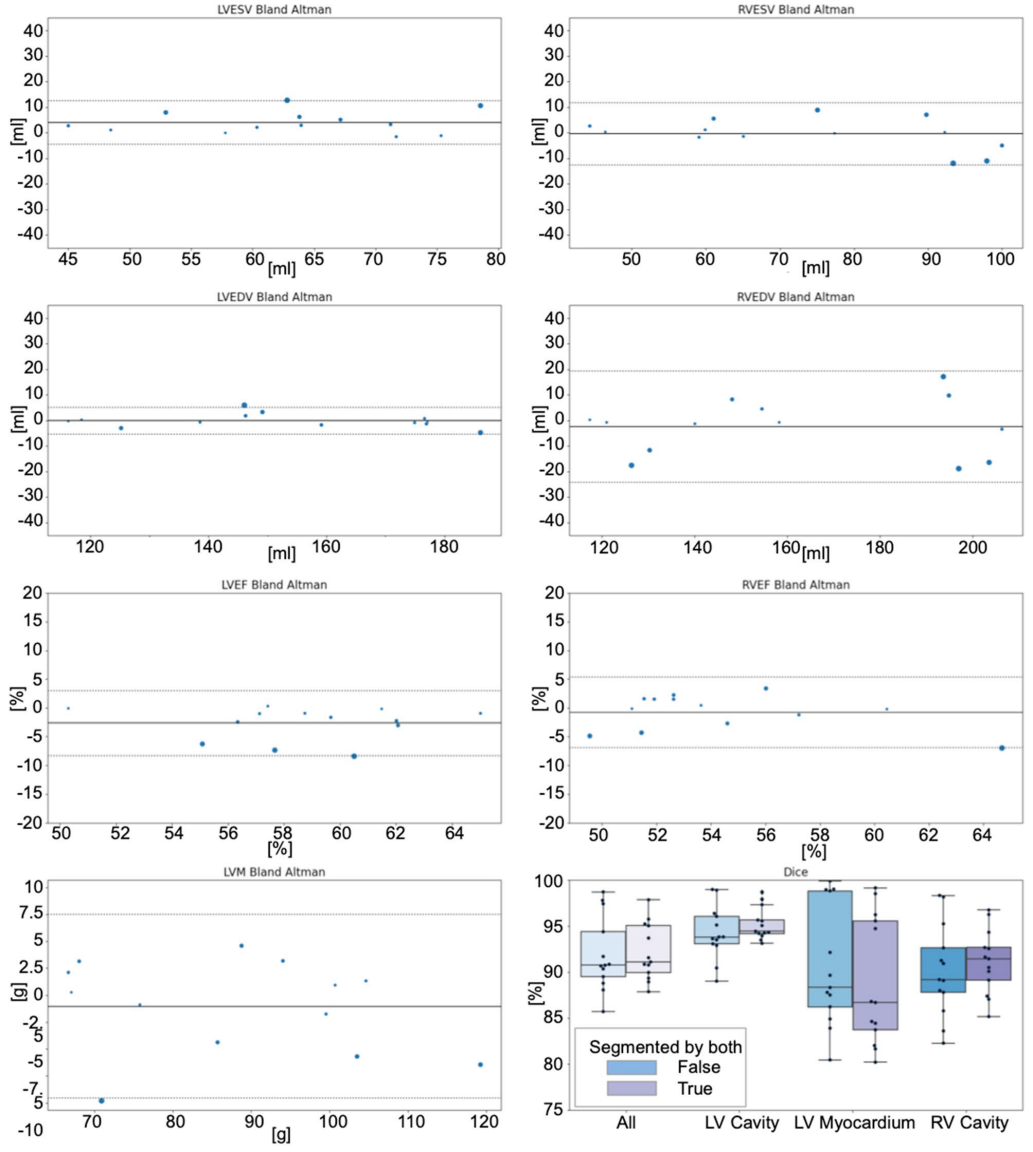
Automatic Generation of Clinical Results Overview. Caption: This plot is automatically generating after loading the cases into Lazy Luna’s GUI. Bland-Altman plots show clinical result averages and differences as points for all cases. Point size represents difference size. The solid line marks the mean difference between readers; the dashed lines mark the mean differences ±1.96 standard deviations. The last plot offers two Dice boxplots per contour type, one for all images, another restricted to images segmented by both readers. The clinical result differences hover around zero for the LV and the RV. The variance is larger for results concerning the RV. Dice values are higher for the LV cavity than for LV myocardium or RV cavity. GUI: Graphical user interface, RV: Right ventricle, LV: Left ventricle, ESV: End-systolic volume, EDV: End-diastolic volume, EF: Ejection fraction, LVM: Left ventricular mass, Dice: Dice similarity coefficient.

Figures are classes that inherit their behaviour from the Python package Matplotlib^28^. Matplotlib figures allow for creating professional static and interactive visualizations. Seaborn^29^ (a wrapper Python package around Matplotlib) is used for statistical visualizations (Fig. 2). Tables are classes that extend Pandas DataFrame objects. Pandas^30^ allows for extensive data analysis and easy storing of spreadsheets, extensive tabular information transformation and data manipulation.

**Figure 3:**
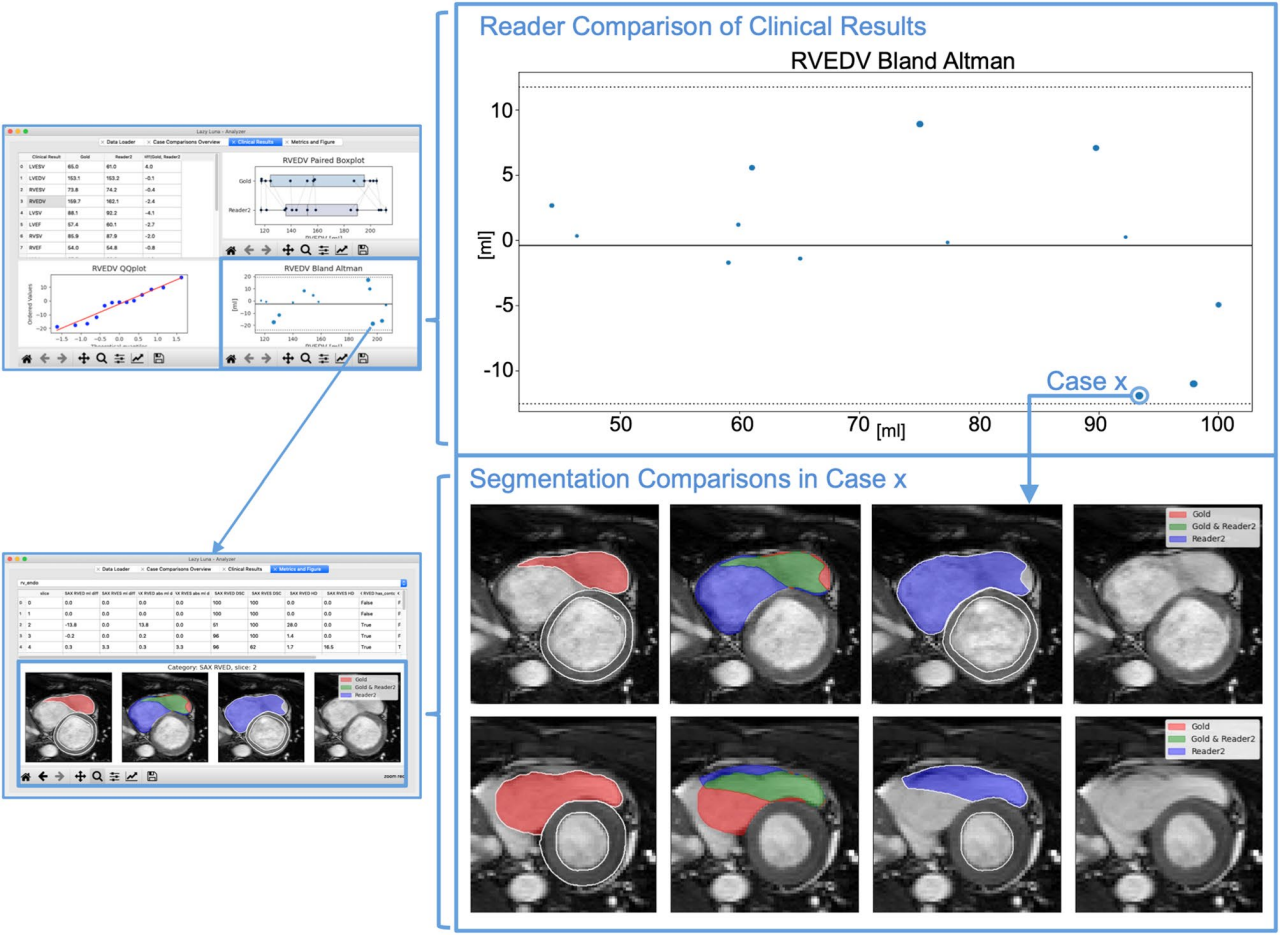
Tracking and Visualizing Reader Differences with Lazy Luna’s GUI. Caption: On the left, two tabs of Lazy Luna’s GUI are presented. On the right, parts of these tabs are magnified. For the top tab the RVESV Bland-Altman plot (outlined in blue) is magnified. For the bottom tab the visualization of segmentation differences is magnified. The first reader’s segmentations (subplot 1) are in red, the second reader’s are in blue (subplot 3). In the second subplot agreement is in green and areas exclusive to one reader are in that reader’s respective colour. The top tab includes a table of clinical result averages per reader next to their average differences (top left), a QQ-plot (bottom left) and paired boxplots (top right). Clicking a point in the Bland-Altman plot opened the lower tab. This tab’s table presents all metric values concerning the case’s segmentations. RV: Right ventricle, ESV: End-systolic volume, GUI: Graphical user interface, QQ-plot: Quantile-quantile plot.

**Figure 4:**
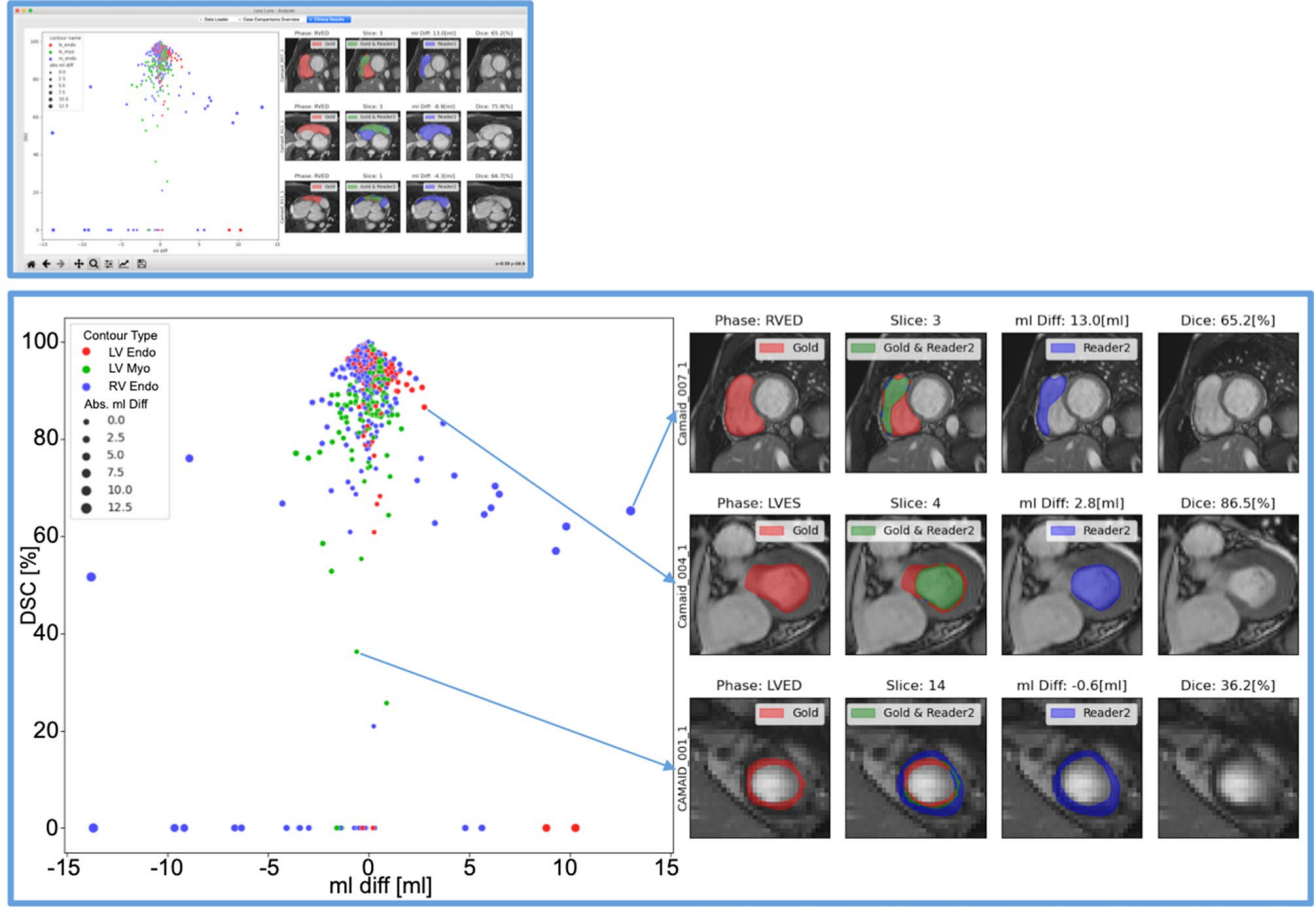
Interactive Correlation Plots of Segmentation Comparisons. Caption: The above window shows the interactive plot in the GUI. Below the plot is enlarged. Every point represents a contour comparison as millilitre difference and Dice value. Its colour distinguishes LV endocardial contour (red), LV myocardium (green) and RV endocardial contour (blue) contours. The point size represents the absolute millilitre difference. On the right visualizations of the comparisons are presented. The arrows show where they were selected from within the correlation plot. GUI: Graphical user interface, RV: Right ventricle, LV: Left ventricle, ES: End systole, ED: End diastole, Endo: Endocardial contour, Myo: Myocardial contour, Abs. ml diff: Absolute millilitre difference.

The graphical user interface (GUI) builds on PyQt5, which has Python bindings to Qt version 5^31^. Matplotlib figures and DataFrames are easy to integrate into PyQt5 GUIs. Interactive Matplotlib figures (Figs. 3, 4) can also be integrated, allowing for tracking function by linking different figures to each other that offer insights on several levels of analysis (such as CRs and metric values, or metric values and qualitative visualizations).

Lazy Luna offers several automated outputs. These include the calculation of tables of metric values (for all phases and slices) for all cases and the calculation of tables of CRs and their differences for all cases (supplementary information). It also produces summary tables for clinical value differences and a metric evaluation of the contours they are based on (Table 1) and for the metric values decomposed by contour type and cardiac position (Table 2). Lazy Luna offers the automatic generation of figures, such as Bland–Altman plots for clinical value distributions and Dice values as boxplots (Fig. 2).

**Table 1:** Title: Reader comparison of clinical results and segmentation metric values. Caption: Clinical result differences between readers are presented in their averages and standard deviations (in blue). They are joined with metric value averages concerning the clinical results above them (in grey). For example: the Dice values below LVEF, LVEDV, LVESV concern the LV cavity. The table presents two Dice values, one for all slices, another restricted to slices segmented by both readers. LV: Left ventricle, LVEF: Left ventricular ejection fraction, Legend: LVEDV: Left ventricular end-diastolic volume, LVESV: Left ventricular end-systolic volume, HD: Hausdorff metric, LVM: Left ventricular myocardial mass, RVEF: Right ventricular ejection fraction, RVEDV: Right ventricular end-diastolic volume, RVESV: Right ventricular end-systolic volume, Std.: Standard deviation.

	Clinical result	Mean	Std
0	LVEF [%]	− 2.6515	2.892012
1	LVEDV [ml]	− 0.1255	2.716635
2	LVESV [ml]	4.009375	4.37966
3	Dice (all slices) [%]	94.28326	2.868816
4	Dice (slices contoured by both) [%]	95.23182	1.746661
5	HD [mm]	0.652106	0.355608
6	LVM [g]	− 1.03389	4.35594
7	Dice (all slices) [%]	90.59014	6.579328
8	Dice (slices contoured by both) [%]	88.8001	6.948619
9	HD [mm]	0.849034	0.523192
10	RVEF [%]	− 0.75374	3.131688
11	RVEDV [ml]	− 2.39193	11.12953
12	RVESV [ml]	− 0.41506	6.187294
13	Dice (all slices) [%]	90.18286	5.009351
14	Dice (slices contoured by both) [%]	91.15519	3.469649
15	HD [mm]	1.628765	0.764461
16	Dice (all slices. all contours) [%]	91.90448	4.006083
17	Dice (slices contoured by both. all contours) [%]	92.15594	3.055814
18	HD (all contours) [mm]	1.082155	0.482872

**Table 2:** Title: Segmentation metric values by contour and cardiac position. Caption: The columns specify the contour type. The sections refer to different cardiac positions (defined by the first reader). The table presents two Dice values, one for all slices, another restricted to slices segmented by both readers. Legend: Midv.: Midventricular, HD: Hausdorff metric, Abs. ml diff.: Absolute millilitre difference.

	Position	Metric	LV Endocardial Contour	LV Myocardial Contour	RV Endocardial Contour
0	basal	Dice (all slices) [%]	87.99322	87.053	71.76934
1	basal	Dice (slices contoured by both) [%]	93.40139	81.29877	74.57156
2	basal	HD [mm]	1.930015	2.130997	8.128019
3	basal	Abs. ml diff. (per slice) [ml]	1.361211	0.937157	3.167208
4	midv	Dice (all slices) [%]	96.91416	91.09024	94.12743
5	midv	Dice (slices contoured by both) [%]	96.12728	89.18645	93.35773
6	midv	HD [mm]	0.835387	0.990279	2.024506
7	midv	Abs. ml diff. (per slice) [ml]	0.307792	0.421006	0.609953
8	apical	Dice (all slices) [%]	83.54174	74.24892	81.71449
9	apical	Dice (slices contoured by both) [%]	83.60426	66.52359	82.93531
10	apical	HD [mm]	0.99975	1.372196	1.579264
11	apical	Abs. ml diff. (per slice) [ml]	0.184053	0.468395	0.234856

### Ethical approval

The local ethics committee of Charité Medical University Berlin gave ethics approval for the original study (approval number EA1/323/15). All patients gave their written informed consent before participating in the study. All methods were carried out in accordance with relevant guidelines and regulations.

## Results

It was possible and feasible to merge the evaluation methods of medical experts and CNN developers. The software automatically structures Dicom images and annotations allowing for comparisons between readers. The cases are compared via their segmentations and CR simultaneously while tracking errors. Calculating all metrics and CRs on the contour level provides sub-pixel accuracy. Lazy Luna can be used to perform inter- and intraobserver analyses. As the software package is described in “Methods” the results section presents Lazy Luna’s GUI and its generated outputs to illustrate a reader comparison performed with Lazy Luna.

### Quantitative results for the use-case

The comparison of the readers’ cardiac function assessments produced the following analysis. The readers show good general agreement on quantitative CRs and segmentation metric values (Table 1). Lazy Luna calculated a CRs spreadsheet (supplementary information), which was used to calculate Pearson’s correlation coefficients for the CRs assessed by both readers. These are LVESV: 91%, LVEDV: 99%, RVESV: 96%, RVEDV: 95%, LVSV: 95%, LVEF: 74%, RVSV 87%, RVEF: 78%, LVM: 97%. Average Dice values are 91.9% for all images and 92.2% for images segmented by both readers. Details are in Table 1. Furthermore, these results can be displayed as single plots to illustrate the result similarities and differences. This is given in Fig. 2, which shows an automatically produced overview of CRs.

### Qualitative results for the use-case

Furthermore, the use-case was also evaluated qualitatively with a visualization of segmentation differences, which was implemented for the GUI. That allows an identification of different slice selection or interpretation, which may lead to large volume differences. An example of a disagreement is given in Fig. 3.

### Tracking differences in the use-case

CR differences can be caused in different cardiac positions and structures. Lazy Luna can track segmentation differences and their impacts on CRs. For this use-case investigating the cardiac position of segmentation difficulties reveals that the midventricular slices have higher Dice values for all contour types (LV cavity: 97%, LV myocardium: 91%, RV cavity: 94%). As a result millilitre differences remain small in these slices (< 1 ml). Segmentation difficulties are larger in basal and apical slices (Table 2). The Dice metric is poorest for the LV myocardium in the apical slices (74%). However, the impact in clinical values is smaller because the millilitre differences remain small (< 0.5 ml). The Dice metric values are also lower in the basal slices (LV cavity: 88%, LV myocardium: 87%, RV cavity: 72%) (Table 2, Fig. 3). However, the millilitre differences are larger in the basal slices, especially those concerning the RV (> 3 ml, Table 2), which causes larger millilitre differences in the CRs. One of Lazy Luna’s interactive GUI tabs allows for exploring this phenomenon (Fig. 4). An interactive metrics correlation plot shows that RV endocardial segmentation disagreements produce the largest RV millilitre differences and provides visualizations of selected differences.

## Discussion

Our main achievement is the implementation of the investigative software Lazy Luna, which is capable of performing a multilevel analysis on reader differences with a graphical user interface. The functionality of Lazy Luna was illustrated by carrying out an interobserver analysis between two experienced readers. This analysis allowed for elucidating segmentation differences in order to give a detailed description of reader differences for short-axis cine images.

Backtracking CR differences in Bland–Altman plots to visualizations of segmentation differences indicated that major millilitre differences might accumulate in basal slices. Correlation plots of all metric values offered insights into qualitative reasons for RV endocardial contour disagreements. It also provided visual confirmation of the RV being difficult in the basal slices and a common cause for larger millilitre differences in CRs. The tabular metric values provided further quantitative evidence for basal slices causing the largest millilitre differences, although the apical slices are similarly difficult to segment accurately.

Furthermore, it is expected, that Lazy Luna could be helpful as a tool for CNN developers and medical experts alike. It allows for streamlining the comparison of readers in a fashion that satisfies both communities. Lazy Luna calculates accurate CRs and metric values, automatizing error-prone and time-intensive spread sheet generation. Interactive visualizations allow for understanding differences on several levels of analysis as well as suggest causal relationships between segmentation failures and CR outliers.

The Dice metric and the Hausdorff distance were taken from the surrounding literature in CNN development^9,13,14,16,32^. Two different methods were used for calculating average Dice metrics, one value concerns all images, the other concerns only images segmented by both readers. In literature it is often unclear how the Dice metric values are averaged over cases and both considerations capture relevant aspects of the segmentation task^16,33^. The metrics were extended to include the millilitre difference for the medical community, which is usually more interested in the impact of segmentation choices on volume differences.

These metrics could be arbitrarily expanded to meet other needs. Several other metrics can also be found in the surrounding literature such as the Intersection over Union^19^ or the Average Surface Distance^9,17^, which could be implemented accurately to apply to Shapely objects.

Pre-processing images for Lazy Luna requires manual selection due to the lack of common image-type identifiers among vendors and sequence types. Lazy Luna currently semi-automates this by presenting the user all images concerning a case in a table grouped by Dicom tags (including seriesDescription, seriesInstanceUID and annotations by group) so that the relevant images can be selected manually. In literature, several machine-learning supported image classification methods have been experimented with to automate this task^34,35^. Pre-processing should be simplified in the future by assisting the user with automated suggestion of image types.

Training readers in CMR as well as in other fields includes curriculum-based education, simulation and competency assessment^6,36,37^. One-on-one teaching with immediate feedback is considered most effective^37^. The relevance of training has been shown to increase the quality of LV volume evaluation^6,38^. However, this type of training requires time intensive training sessions with a teacher present who explains many cases directly. That could be supported by Lazy Luna as the fast and automatic comparison of two readers may help to improve the training of trainees without direct coaching including significant time investment for manual evaluation and to bring support in the place in which additional coaching is required.

Furthermore, CNNs play an increasing role in CMR post-processing. Several confounders can complicate the automatic segmentation of images. Generalizing over different datasets can be difficult. Confounders include: different sequences such as the short-axis cine images in this paper^5,39,40^, different scanners^19^, different pathologies^17^ (i.e. LV and RV hypertrophies) and artefacts that must be identified and excluded before automatic segmentation^1^. Lazy Luna offers functionality for the calculation of inter- and intraobserver comparisons for the assessment of segmentation accuracy.

CNNs should be compared to readers on a contour level for precise evaluation. Several CMR segmentation contests include sophisticated evaluations for segmentation quality and CRs. However, they disregard the inaccuracy caused by comparing on pixelated segmentation masks as ground truth segmentations instead of comparing contours as polygons^16,41^.

CNN training procedures could integrate Lazy Luna’s capabilities as part of the training procedure. By storing the annotations for the evaluation dataset in Lazy Luna’s format, Dice metric values would be offered, but clinically relevant outliers of cases would also be analysed accordingly. This would enhance the evaluation by considering the interconnected nature of Dice metric values and the volumetric differences they cause.

In several guidelines it is recommended to perform evaluation based on the AHA model^1^. In the future, Lazy Luna will provide the AHA model as an intermittent analysis step, allowing for tracking of annotation differences from AHA-segments.

The classes generically keep track of images and annotations. This software backend can be extended to include other quantification techniques as well.

### Limitations

Lazy Luna is intended to be generic, however currently it is limited to short-axis cine stacks and should be shown to generalize to other cardiac structures and imaging sequences. Other outputs such as AI segmentations maps and other software vendors are to be tested in future work.

Lazy Luna is intended to be open-source in the future as to be available to and extendable by other researchers. Other image and annotation pre-processing steps (i.e. steps typically necessary for AI-contests) will be automatically addressed before source-code publication so that researchers can reproduce results on available segmentation contests.

## Conclusion

The introduced software Lazy Luna enables an automatic multilevel evaluation of readers on quantitative results. In our use-case the readers showed an overall good agreement on the level of individual segmentations and clinical results. Lazy Luna allowed pinpointing origins of large millilitre difference to segmentation differences in specific cardiac structures and locations. Future developments include generalizing the software’s applicability to different sequences and anatomical structures.

## Supplementary Information

Supplementary Information 1.

Supplementary Information 2.

Supplementary Information 3.

Supplementary Information 4.

Supplementary Information 5.

## Data Availability

The datasets analysed during the current study are not publicly available due to patient data privacy but are available from the corresponding author on reasonable request after communication with the legal department as there are special rules based on the EU law and the rules of the Berlin data officer rules. The datasets generated during this study are included in this published article and its supplementary information files.
